# Unexpected Encounter: A New Genus of Orthosiini (Noctuidae: Hadeninae) Revealed by Tit Predation in Late-Winter Baihuashan National Nature Reserve, Beijing

**DOI:** 10.3390/insects17010121

**Published:** 2026-01-21

**Authors:** Jun Wu, Nan Yang, László Ronkay, Hui-Lin Han

**Affiliations:** 1 College of Forestry, Northeast Forestry University, Harbin 150040, China; wujun5911@foxmail.com; 2 Northeast Asia Biodiversity Research Center, Northeast Forestry University, Harbin 150040, China; 3 Beijing Baihuashan National Reserve, Beijing 102461, China; straybird726@163.com; 4 Heterocera Press Ltd., Szent István körút 4, H-1137 Budapest, Hungary; laszlo.ronkay2@gmail.com; 5 Key Laboratory of Sustainable Forest Ecosystem Management, Ministry of Education, Northeast Forestry University, Harbin 150040, China

**Keywords:** avian predation, DNA barcodes, morphology, new genus, new species, owlet moth, ecological observations, taxonomy

## Abstract

During a late-winter field survey in Baihuashan National Nature Reserve, Beijing, several noctuid moths were observed flying during the daytime at low temperatures and being actively preyed upon by Marsh tits, which removed the heads and wings of captured individuals. These observations indicate that adults of this noctuid lineage are active in late winter, providing a critical nutritional resource for insectivorous birds during the ecologically constrained, food-limited winter period. Here, we formally describe this lineage as a new genus, *Shoudus* gen. nov., based on a new species, *S. baihuashanus* sp. nov., collected from Baihuashan reserve, including three specimens retrieved during active interception of tit predation, along with detached wings and heads recovered from the snow. The new genus is placed in the tribe Orthosiini Guenée, 1837, primarily based on adult external morphology, including large compound eyes with long interfacetal hairs and bipectinate male antennae, as well as forewing patterning similar to certain orthosiine genera such as *Perigrapha* and *Clavipalpula*. Notably, the dark reddish-brown forewings with sharply contrasting pale markings, as seen in the new genus and these related genera, appear well adapted for camouflage against bark, leaf litter, and exposed soil in their habitats—potentially functioning as both background matching and disruptive coloration. To further assess its phylogenetic placement, we conducted a molecular analysis based on mitochondrial COI sequences (13 newly generated and 6 retrieved from BOLD/NCBI). The resulting maximum likelihood and Bayesian trees consistently support the monophyly of the new genus and reveal a close phylogenetic relationship with *Orthosia*, the type genus of Orthosiini. This integrative evidence strongly supports the recognition of *Shoudus* as a distinct lineage within Orthosiini.

## Simple Summary

During late-winter surveys in northern China, we observed a previously undocumented moth flying in daytime at low temperatures and being preyed upon by Marsh Tits. Based on these observations and collected material, we describe a new genus and species, *Shoudus baihuashanus*. Morphological characters and DNA data place this lineage in the tribe Orthosiini Guenée, 1837 and indicate a close relationship with *Orthosia*. This discovery highlights a poorly known group of moths active in late winter and suggests that such insects may contribute to the winter diet of insectivorous birds.

## 1. Introduction

In temperate forest ecosystems, overwintering and late winter-early spring emerging insects represent one of the few prey resources available to insectivorous birds during food-scarce winter months, playing a critical ecological role. Previous studies have shown that, even under harsh conditions, insectivorous birds—particularly species of Paridae—actively prey on overwintering Lepidoptera adults and larvae (Kirk et al., 1996 [1]). Long-term monitoring in northern forests further indicates that the late-winter emergence of moths benefits the breeding success of both resident and migratory birds. In northern boreal regions, this bottom-up effect is reflected in a positive correlation between the biomass of moths—especially those overwintering as adults, pupae and eggs—and the biomass of forest birds that rely heavily on caterpillars to feed their nestlings (Yazdanian et al., 2024 [2]).

Meanwhile, studies on moth camouflage have demonstrated that their wing coloration and pattern often evolve to match background substrates such as tree bark, leaf litter, or snow-covered soil, thereby reducing detection by visual predators (Stavenga et al., 2020; Kang et al., 2015; Walton and Stevens, 2018 [3,4,5]). These observations highlight that moths active in late winter constitute a group of considerable biological importance. However, in the Palearctic region, such ecological interactions—particularly those involving late-winter-active noctuid adults—remain poorly understood.

The tribe Orthosiini Guenée, 1837 is a diverse group of “winter Noctuidae” primarily distributed in the temperate Palaearctic region; most species are univoltine and inhabit forested ecosystems, with adults typically active in early spring or late autumn (Ronkay et al., 2010 [6]). During recent field surveys in Baihuashan Reserve, Beijing, China, several specimens belonging to a previously unrecognized orthosiine lineage were collected. Notably, the discovery was facilitated by observing the predation of these moths by tits (Paridae), which drew our attention to this late-winter-active species. Preliminary morphological examination revealed a unique combination of adult and genital characters, including relatively small body size, very long rami of bipectinate male antennae, uniformly colored fore- and hindwings, and a simplified male genital structure. Phylogenetic analysis based on mitochondrial COI barcode data further supported the distinctiveness of this lineage from all known genera currently placed within Orthosiini.

In this study, we describe a new genus and species of Orthosiini from northern China based on combined morphological and molecular evidence. Diagnostic characters of the new genus are compared with related genera such as *Perigrapha* Lederer, 1857, *Clavipalpula* Staudinger, 1892, and *Lasianobia* Hampson, 1905, and its taxonomic placement is discussed. Given the limited resolution and sometimes unstable topologies inferred from single-locus mitochondrial datasets, we advocate for future incorporation of nuclear gene markers to better resolve generic boundaries within Orthosiini. In addition, we briefly document the ecological characteristics observed during field collection, including the occurrence of adults in late winter, predation by passerine birds, and putative adaptive features of wing coloration.

## 2. Material and Methods

### 2.1. Taxon Sampling and Comparative Rationale

The specimens examined in this study were primarily collected in China. For newly collected material, three legs from one side of each individual were removed for DNA extraction and preserved in 100% ethanol at −20 °C. For phylogenetic reconstruction, two noctuid species—*Himalaea unica* Hreblay & Ronkay, 1998 (Amphipyrinae: Psaphidini) and *Ulochlaena hirta* (Hübner, [1813]) (Xyleninae: Episemini)—were selected from BOLD (Barcode of Life Data Systems, Guelph, Canada, https://v4.boldsystems.org/, accessed on 28 February 2025) and NCBI (https://www.ncbi.nlm.nih.gov/nucleotide, accessed on 23 December 2024) databases as outgroups. These taxa were chosen due to the partial resemblance of their male genitalia to that of the new genus (Ronkay et al., 2001 [7]; Saldaitis et al., 2022 [8]).

To assess the taxonomic placement of the new genus within Orthosiini, we included both newly generated sequences, including that of the new species, and several publicly available sequences. These represent the genera *Orthosia* Ochsenheimer, 1816, *Clavipalpula*, and *Perigrapha*, all members of Orthosiini, as well as *Lasianobia*, a member of Hadenini. Morphologically, *Lasianobia*, *Clavipalpula*, and *Perigrapha* are more or less similar to the new genus, especially in having forewings with irregular patterns marked by sharp contrasts between light and dark areas (Saldaitis et al., 2012, 2018; Aulombard et al., 2020 [9,10,11]). The genus *Orthosia* was included because it is the type genus of the tribe Orthosiini, and we selected its type species, *O. incerta* (Hufnagel, 1766), for molecular analysis (Wiesmair et al., 2020 [12]).

*Hadena eximia* (Staudinger, 1895) and *Niaboma xena* (Staudinger, [1896]) were incorporated because they belong to Hadenini, a tribe closely related to Orthosiini, and share forewing pattern elements that are partially similar to those of *Perigrapha* (Gyulai and Ronkay 2001 [13]; Staudinger 1896 [14]). Detailed collection localities, BOLD sample IDs, and GenBank accession numbers are provided in Table 1. In addition to newly generated sequences, we included one sequence labeled as *Lasianobia superba* (Alphéraky, 1892) (accession no. MH722409) from NCBI for phylogenetic analysis. After examining the holotypes of both *L. superba* and *L. dichelostigma* (Tams, 1929), and considering their respective distribution ranges (the true *L. superba* occurs in the Kuku Noor region of Qinghai, China, while *L. dichelostigma* inhabits southern Tibet (China), Nepal, Sikkim, Bhutan, etc.), we consider the NCBI sequence labeled as *L. superba* to be a misidentification of *L. dichelostigma*. However, in this study, we retain the original name *superba* as annotated in the database.

All sequences of the new species and the newly sequenced data presented in this paper have been deposited in GenBank under Accession Numbers PX069813–PX069825 (Table 1).

### 2.2. Morphological Study

**Figure 1:**
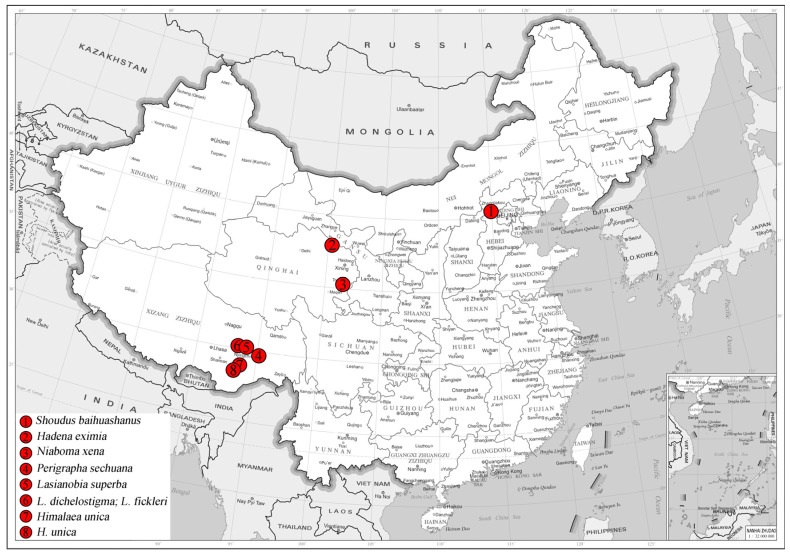
Specimens collecting sites of newly sequenced species from China in this study: (**1**) Baihuashan Reserve, Beijing; (**2**) Qingyanggou, Mt. Qilian Reserve, Qinghai; (**3**) Xiuxiangou, Huangnan Autonomous Prefecture, Qinghai; (**4**) Gu Township, Bomi County, Xizang; (**5**) East of Mt. Sejila, Linzhi County, Xizang; (**6**) West of Mt. Sejila, Linzhi County, Xizang; (**7**) Lang Town, Linzhi County, Xizang; (**8**) Dengmu Township, Linzhi County, Xizang.

The type series was collected in Beijing, China, by netting during the daytime. Standard methods for dissection and preparation of the genitalia slides were used. The specimens were photographed using a Canon M6 Mark II camera (Canon Inc., Tokyo, Japan), whereas the genitalia slides were photographed with an AOSVI Hk-830 microscope (Shenzhen Aoswei Optical Instruments Co., Ltd., Shenzhen, China) aided by Helicon Focus 7 software (Helicon Soft Ltd., Kharkiv, Ukraine) and further processed in Adobe Photoshop CS6 (Adobe Inc., San Jose, CA, USA). Terminology of genitalia follows Kononenko and Han (2007) [15]. The map of collecting sites is list in Figure 1.

All type materials of the new species are deposited in the collection of the Northeast Forestry University (NEFU), Harbin, China.

### 2.3. DNA Extraction and PCR

Standard DNA extraction and amplification methods were performed. Genomic DNA was extracted from three legs of each specimen using the TaKaRa MiniBEST Universal Genomic DNA Extraction Kit Ver. 5.0 (Takara Bio Inc., Shiga, Japan), following overnight incubation at 56 °C and according to the kit’s protocol. Polymerase chain reactions (PCRs) were conducted on a PCR Thermal Cycler (Hangzhou LongGene Scientific Instruments Co., Ltd., Hangzhou, China) using the primers LCO1490 and HCO2198 (Folmer et al. 1994 [16]) to amplify a 658 bp fragment of the mitochondrial cytochrome coxidase I (COI) gene.

The total reaction volume was 25 μL, consisting of 0.5 μL template DNA, 10 μL ddH_2_O, 12.5 μL 2× Rapid Taq Master Mix (Vazyme Biotech Co., Ltd., Nanjing, China), and1 μL each of forward and reverse primers (synthesized by Sangon Biotech from Changchun, China). The PCR program included an initial denaturation at 95 °C for 3 min; 35 cycles of 95 °C for 30 s, 55 °C for 30 s, and 72 °C for 1 min; with a final extension at 72 °C for 5 min.

### 2.4. Molecular Sampling and Phylogenetic Methods

A total of 19 COI barcode sequences were aligned using MAFFT v7.490 (Katoh and Standley, 2013 [17]) with the L-INS-i algorithm implemented in PhyloSuite v1.2.3 (Zhang et al., 2020 [18]). The alignment was subsequently trimmed using trimAl v1.4 (Capella-Gutiérrez et al., 2009 [19]) under the “automated1” mode to remove poorly aligned regions. The final alignment was used for phylogenetic tree reconstruction under both Maximum Likelihood (ML) and Bayesian Inference (BI) frameworks.

For the ML analysis, IQ-TREE v2.2.0 was employed (Nguyen et al., 2015 [20]) under the K80+G4+F model for 1000 ultrafast (Minh et al., 2013 [21]) bootstraps, approximate Bayes test (Anisimova et al., 2011 [22]), as well as the Shimodaira–Hasegawa–like approximate likelihood-ratio test (Guindon et al., 2010 [23]). The tree was rooted using two noctuid species outside Orthosiini as outgroups.

For the BI analysis, we used MrBayes v3.2.7a (Ronquist et al., 2012 [24]) with the GTR+G+F model. Two independent runs of 2,000,000 generations were performed, with the first 25% of the sampled trees discarded as burn-in. Convergence was assessed by ensuring the average standard deviation of split frequencies was below 0.01 and that the potential scale reduction factor (PSRF) approached 1.00. All estimated sample size (ESS) values, examined using Tracer (University of Edinburgh, Edinburgh, UK), exceeded 200, indicating sufficient sampling of the posterior distribution. Node support was assessed using posterior probability values.

Finally, genetic distances between selected known species and the newly described species were calculated using MEGA 12 (Kumar et al., 2024 [25]). The analysis employed the Kimura 2-parameter model (K2P), including both transitions and transversions. Substitution rate variation among sites was modeled with a gamma distribution (Gamma Distributed, G) with a shape parameter of 0.30. The evolutionary pattern among lineages was assumed to be homogeneous. Missing data were treated using the pairwise deletion method, and all codon positions (1st, 2nd, 3rd) as well as noncoding sites were included in the analysis.

## 3. Results

### 3.1. Taxonomy and Classification

***Shoudus* Wu, Ronkay & Han, gen. nov.**

Zoobank: urn:lsid:zoobank.org:act:CD5513DD-463D-4E9E-B548-6FFD3266BD0B.

Type species: *Shoudus baihuashanus* Wu, Yang, Ronkay & Han, sp. nov.

Chinese name. 首都夜蛾属.

**Figure 2:**
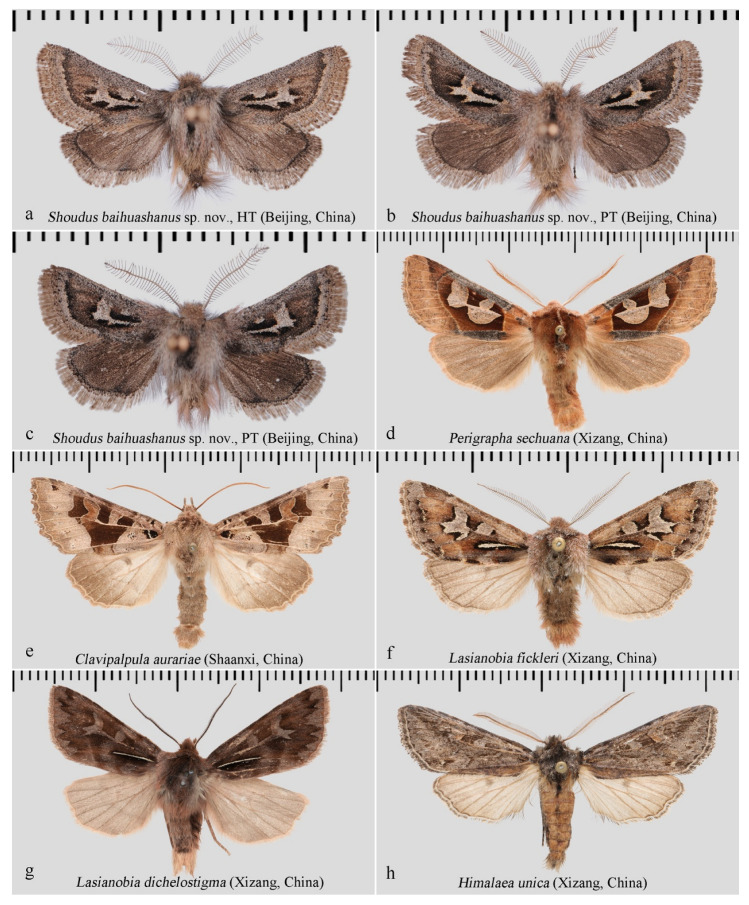
Adult males of *S. baihuashanus* sp. nov. and related species. (**a**–**e**) Hadeninae: Orthosiini; (**f**,**g**) Hadeninae: Hadenini; (**h**) Amphipyrinae: Psaphidini, included for comparison due to similarity in male genitalia. All deposited in NEFU.

**Figure 3:**
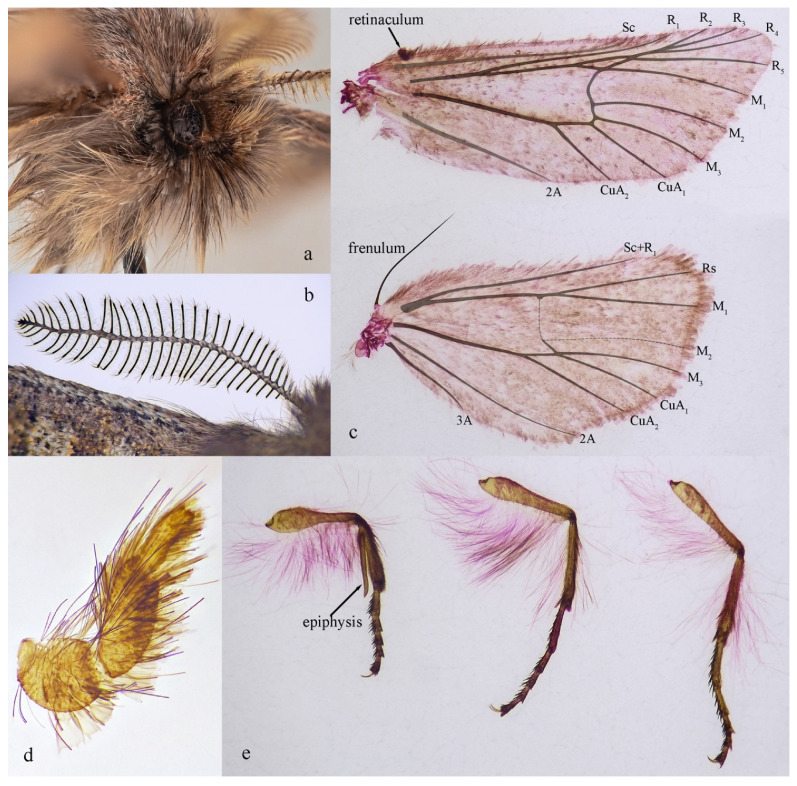
*S. baihuashanus* sp. nov.: (**a**) Eye and labial palpus. (**b**) Antenna. (**c**) Fore- and hindwings. (**d**) Labial palpus. (**e**) Legs.

**Diagnosis:** Externally, the new genus (Figure 2a–c and Figure 3a–e) resembles several other genera within the tribe Orthosiini, including *Perigrapha* (Figure 2d) and *Clavipalpula* (Figure 2e), as well as *Lasianobia* (Figure 2f,g) of Hadenini, all of which typically exhibit a reddish-brown ground color and contrasting forewing patterns. However, the new genus is distinguished by its smaller body size, uniformly colored fore- and hindwings, broadly bipectinate male antennae with exceptionally long and sparsely arranged rami, and the presence of long setae on the thorax and abdominal tip. Most notably, unlike other genera in Orthosiini that possess a well-developed and functional proboscis, the new genus entirely lacks a proboscis. In contrast, species of *Lasianobia*, *Perigrapha*, and *Clavipalpula* tend to be larger in size, with hindwings that are usually paler than the forewings; their male antennae are short-bipectinate or filiform, and the setae on the thorax and terminal abdomen are relatively short.

**Figure 4:**
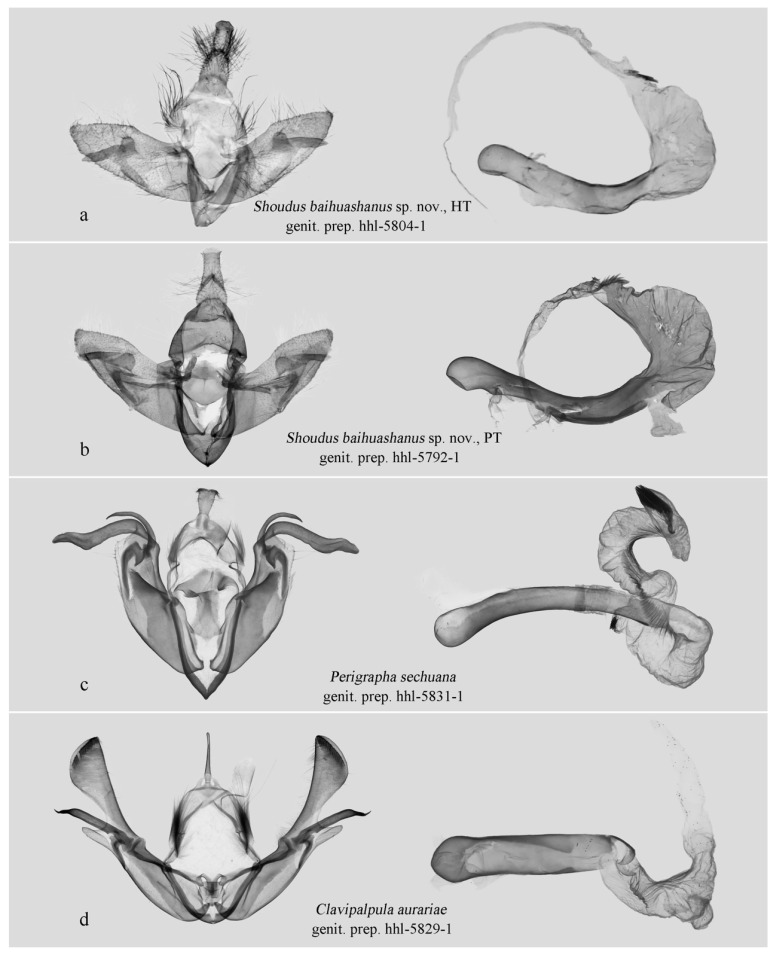
Male genitalia. (**a**–**d**) Hadeninae: Orthosiini. All deposited in NEFU.

**Figure 5:**
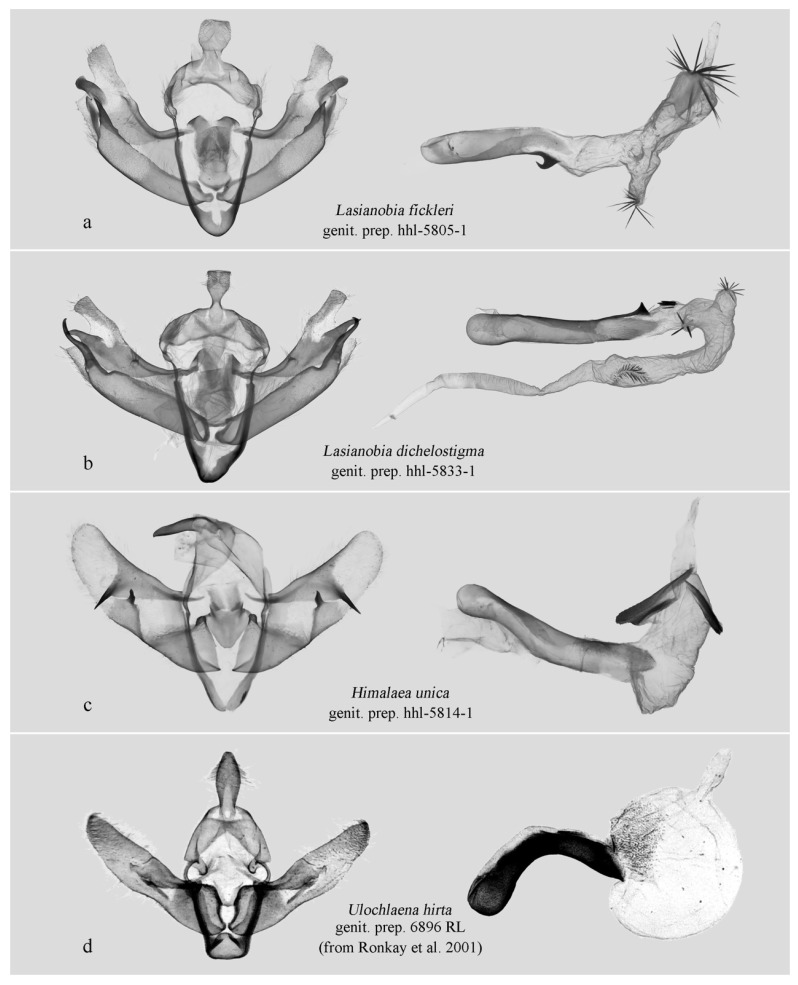
Male genitalia. (**a**,**b**) Hadeninae: Hadenini; (**c**) Amphipyrinae: Psaphidini; (**d**) Xyleninae: Episemini. (**c**,**d**) are presented for comparative purposes due to some resemblance of their male genitalia to that of the new species. Deposited in NEFU except (**d**) (from Ronkay et al. 2001 [7]).

In the male genitalia, the new genus is characterized by a simplified valva lacking a ventral process, no distinct constriction (“neck”) between the cucullus and the main body of the valva, and a markedly inflated vesical (Figure 4a,b). In contrast, *Perigrapha* (Figure 4c), *Clavipalpula* (Figure 4d), and *Lasianobia* (Figure 5a,b) typically possess a saccular process on the valva, a distinct “neck” region, and a more tubular vesica.

The male genitalia of the new genus show some similarity to those of *Himalaea unica* (Figure 2h and Figure 5c) and *Ulochlaena hirta* (Figure 5d), particularly in their overall simplicity and the presence of a conspicuously swollen vesica apex. However, the external appearance of the new genus is clearly distinct from *H. unica*. While it superficially resembles *U. hirta*, the two can be easily distinguished by the clarity of their wing patterns: in *U. hirta*, the forewing venation is prominently visible, and the antemedial line is distinct, the boundaries of the orbicular and reniform spots are sharply defined. In contrast, the forewing patterns of the new genus are significantly more diffused.

**Description: Adult (male).** Eyes large, rounded, with long interfacetal hairs (Figure 3a). Antennae broadly bipectinate, rami long and extending from base to apex and covered with long, dense lateral, apically fasciculate cilia (Figure 3b). Labial palpi short, upturned, brown with whitish tips; covered with dense long hairs in addition to scales (Figure 3d). Proboscis absent. Dorsum of thorax and abdomen clothed with dense, long hairs, grayish white to dark reddish brown. Forewing broad, triangular, costa slightly concave; ground color brown; central area with irregular grayish white patch bordered distally with dark brown; subterminal line distinct, forming a diffuse dark brown band. Hindwing similar in color to forewing; terminal line with dark brown. Male frenulum long, approximately one-third length of hindwing costa. Fore- and hindwing fringes long. **Forewing venation** (Figure 3c, upper): Sc closely alongside R_1_ near distal section; R_2–4_ stalked, R_4_ arising before R_2_; R_5_ from upper corner of discal cell, joining R_2–4_ stalk beyond cell to form a small accessory cell; M_1_ and R_5_ arising from same point; M_2_ separate from M_3_; Cu straight; 1A + 2A slightly curved medially. **Hindwing venation** (Figure 3c, lower): Sc + R_1_ slightly curved inward; Rs and M_1_ shortly stalked; M_3_ and CuA_1_ from lower corner of discal cell; 2A remote from CuA_2_, slightly curved outward; 3A close to anal margin. **Legs** (Figure 3e): Tibial spurs 0–2–4; femora and tibiae covered with long brown hairs; tarsi with dense short setae; fore tibia with well-developed epiphysis.

**Male genitalia.** Overall structure simpler than that of *Lasianobia*, *Perigrapha*, and *Clavipalpula*. Uncus short, stout, apically blunt. Tegumen broad. Valva sword-shaped, gradually tapering distally; valva with clasper–ampulla complex; ampulla a rounded, hump-like process on inner side; clasper long, directed outward, with basal plate forming triangular lobe; fine but distinct narrow connection present between clasper and distal end of sacculus. Juxta broad; Vinculum robust; saccus V-shaped. Aedeagus tubular, slightly curved; vesica swollen, bearing a cluster of cornutus at apex.

**Female genitalia**. Unknown.

**Etymology:** The generic name *Shoudus* is derived from the pinyin transliteration of “Shoudu (首都)”, the Chinese term for “capital,” referring to Beijing, where the new genus was discovered. The name is treated as masculine in gender.

**Remark**: This genus is known to be a monotypic genus and is found only in a nature reserve in Beijing, China.

***Shoudus baihuashanus* Wu, Yang, Ronkay & Han, sp. nov.**

Zoobank: urn:lsid:zoobank.org:act:0B831599-2519-4BA9-9AB0-EEC326DC8626.

Figure 2a–c, Figure 3a–e and Figure 4a,b.

Chinese name. 首都夜蛾.

**HOLOTYPE**: ♂, CHINA, Beijing City, Mentougou Dist., Baihuashan National Nature Reserve, 7 II 2024, Nan Yang leg., genit. prep. hhl-5804-1 [NEFU]. **PARATYPES**: 2 ♂, same data as for holotype, genit. prep. hhl-5792-1 [NEFU].

**Diagnosis:** As outlined in the generic diagnosis, *S. baihuashanus* sp. nov. resembles certain species of *Perigrapha*, *Clavipalpula*, and *Lasianobia* in facies, particularly sharing the general forewing pattern with *Lasianobia*. However, it can be readily distinguished by its smaller body size, the complete absence of a proboscis, long, wooly vestiture of thorax and abdomen, overall habitus, the structure of the male bipectinate antennae, and the distinctive male genitalia.

**Description: Male** (Figure 2a–c and Figure 3a–e). Forewing length 9–10 mm; wingspan 21–23 mm (*n* = 3). Head dark reddish brown. Antennae bipectinate throughout, branches long and sparse, each bearing dense sensilla; apical branches with one (rarely two) long spine-like setae. Eyes large, rounded, with long interfacetal hairs. Labial palpus grayish brown, relatively short, apically upturned, densely covered with long hairs in addition to scales. Proboscis absent. Thorax robust; abdomen short; dense, long hairs present from thoracic dorsum to abdominal tip, varying from grayish white to dark reddish brown. Femur and tibia covered with dense long reddish-brown hairs. Foreleg with well-developed epiphysis, heavily sclerotized, about equal in length to tibia.

Forewing broad, triangular, costa slightly concave; ground color dark brown; costal margin grayish brown; outer margin slightly tinged with grayish white, medial area blackish. Subterminal line distinct, dark brown, band-shaped, nearly parallel to outer margin; other transverse lines inconspicuous. A gradually widening grayish white wedge-shaped patch extending from base of discal cell to median area; projections of this patch extend distally along veins M_3_, CuA_1_, and CuA_2_ in spine-like extensions, with golden-yellowish scales scattered along the margin. Orbicular spot grayish white, oval; reniform spot elongate, elliptical, broadly joined to wedge-shaped patch posteriorly; both spots poorly defined anteriorly, blending with pale costal patch. Fringes long, reddish brown. Hindwing ground color dark brown, veins dark brown; terminal line thick, dark brown; discal spot absent; fringes long, dark reddish brown with intermixed dark brown.

**Male genitalia** (Figure 4a,b). Uncus short, stout, slightly swollen basally, apically flat, densely setose laterally. Tegumen broad, triangular, penicular lobes with tufts of long, removable hairs. Valva simple, sword-shaped. Costa with a strongly sclerotized ridge-like projection basally; median part slightly elevated; digitus absent. Sacculus slightly sclerotized basally, bearing a small tooth-like process present distally. Ampulla shortly hump-like, projecting dorsad, apically setose with short sensory setae; clasper long, sclerotized, its erect part finger-like, projecting ventrad distally. Basal plate of clasper straight, flap-like, sparsely setose, fused partly with distal end of sacculus. Juxta flattened, pentagonal, rather shield-like. Vinculum robust, strongly sclerotized. Saccus V-shaped. Aedeagus slender, tubular, slightly curved medially; carina sclerotized, without carinal spine; vesica swollen, sac-like, everted forward and curved strongly ventrad, bearing a cluster of variably long, fine cornuti apically.

**Distribution**: China (Beijing).

**Bionomics**: *S. baihuashanus* sp. nov. adults were observed flying during midday under low ambient temperatures (ca. −7~11 °C) in late February and early March in Baihuashan and adjacent areas in western Beijing. The habitat consists of temperate deciduous and mixed forests dominated by leafless broadleaved trees.

**Etymology**: The specific epithet *baihuashanus* refers to the Baihuashan Nature Reserve in Beijing, which is the type locality of the new species. The name is formed as a Latinized adjective in the masculine gender to agree with the generic name.

### 3.2. Molecular Phylogenetic Analysis

**Figure 6:**
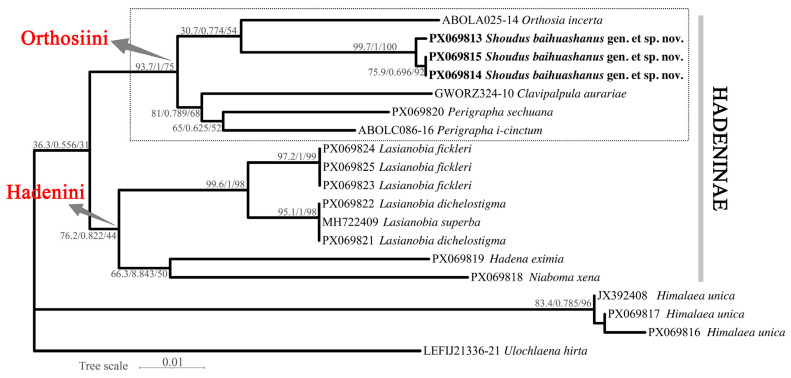
Maximum likelihood tree inferred based on DNA barcode sequences (COI), rooted on two non-Hadeninae species as outgroups. Node support is indicated as SH-aLRT support/Bayesian posterior probabilities/UFBoot values.

The ML tree inferred using IQ-TREE v2.2.0 (Figure 6) recovered the three individuals of the new species (PX069813, PX069814, and PX069815) as a highly supported monophyletic clade (SH-aLRT/Bayesian support/UFBoot: 99.7/1.00/100). This clade is sister to a moderately supported lineage comprising *Orthosia incerta*. Within Orthosiini, *Perigrapha* and *Clavipalpula* also cluster closely with the new genus and *O. incerta*, forming a well-supported clade. Another distinct lineage consists of *Lasianobia* spp., *Niaboma xena*, and *Hadena eximia*, representing members of Hadenini. The outgroup taxa (*Himalaea* and *Ulochlaena*) form a separate lineage outside the Hadeninae cluster.

**Figure 7:**
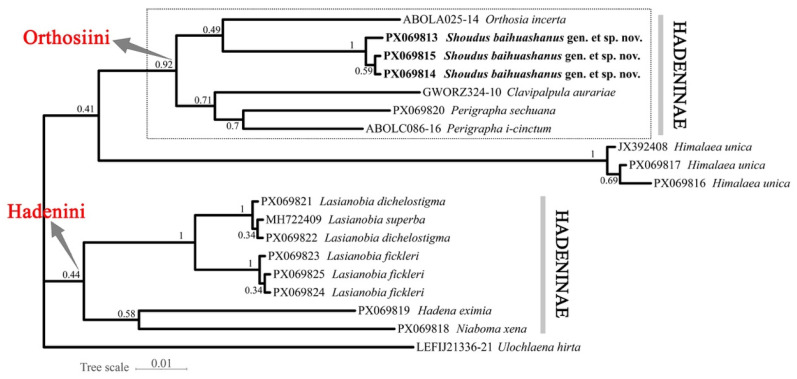
Bayesian inference tree inferred based on DNA barcode sequences (COI). Numbers at nodes indicate PP values.

The BI tree (Figure 7) generated in MrBayes produced an identical topology to that of the ML analysis. The new species group forms a well-supported monophyletic clade (posterior probability = 1.00) and occupies the same sister-group relationship with *Orthosia*. The placement of other major genera and the outgroup taxa is also congruent with the ML tree excepting the *Himalaea* clade.

As shown in the dashed boxes in Figure 6 and Figure 7, the clade containing *S. baihuashanus* exhibits identical topologies in both the ML and BI trees, indicating strong phylogenetic consistency and supporting the monophyly of major clades within Orthosiini. Notably, the three sequences corresponding to the newly proposed genus (PX069813, PX069814, and PX069815) form a strongly supported monophyletic group in both analyses, clearly separated from other genera in the tribe, including *Orthosia*, *Perigrapha* and *Clavipalpula*. This strongly supports the distinctiveness of the new genus at the molecular level.

However, certain topological differences are observed between the ML and BI trees. In the BI tree, one outgroup taxon, *Himalaea unica* (Amphipyrinae: Psaphidini), is erroneously nested within the Orthosiini clade, whereas the ML tree correctly places it outside Hadeninae. This discrepancy likely reflects the limited phylogenetic resolution of mitochondrial COI for deep divergences, particularly in outgroup placement. Considering the overall congruence between the two trees and the morphology-based expectation for *Himalaea*, we interpret its BI placement as an artifact rather than a true phylogenetic signal. By contrast, the three genera assigned to Hadenini (*Lasianobia*, *Niaboma*, and *Hadena*) consistently form a distinct lineage in both analyses.

Despite these minor issues, the phylogenetic position of the new genus remains robust across both trees. Its sister relationship to *Orthosia* is well supported, and the clear molecular differentiation, combined with distinctive male genital morphology, justifies its recognition as a separate genus. The consistency of this clade across both analytical frameworks reinforces the validity of the new genus and demonstrates that the observed topological artifacts do not undermine its establishment.

In this study, the COI sequences of the three male specimens of the new genus were nearly identical: two sequences were completely identical, while the third differed by two nucleotide substitutions (at positions 79 and 367), representing an intraspecific divergence of only 0.3%, which falls well within the expected range of intraspecific variation in Lepidoptera.

Among the sampled taxa within Hadeninae, the smallest interspecific genetic distance was observed between the new genus and *Orthosia incerta* (6.2%), while the largest distance was found between the new genus and *Hadena eximia* (9.7%) (Table 2).

### 3.3. Ecological Observations

**Figure 8:**
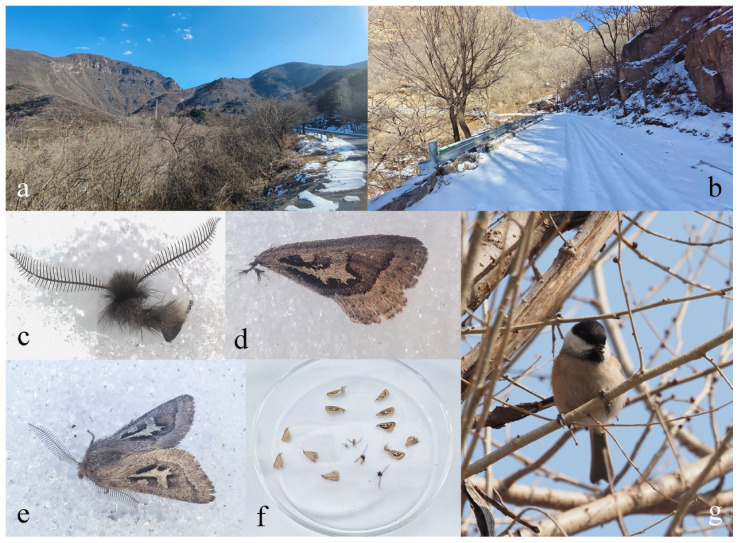
Field observations of *S. baihuashanus* sp. nov. and its ecological context. (**a**,**b**) Winter habitats. (**c**,**d**) Discarded heads and wings of *S. baihuashanus* on snow surface following predation. (**e**) Adult individual of *S. baihuashanus* on snow. (**f**) Collected heads and wings of *S. baihuashanus* from snow. (**g**) Marsh Tit (*Poecile palustris*), a predator of the new species.

During late-winter field surveys across multiple sites in Baihuashan and adjacent areas of Mentougou District, Beijing, adult individuals of *S. baihuashanus* sp. nov. were observed under low-temperature conditions. This species was recorded across multiple years, with all sightings occurring during the late winter months of February or March, when ambient temperatures in the region remain low. At the type locality, midday temperature measurements (around 12:00 p.m.) during sunlit periods reached approximately 6 °C, while shaded areas remained around 2 °C (Table 3). The landscape was largely snow-covered, and surrounding vegetation consisted predominantly of leafless deciduous species, including *Prunus davidiana* (Rosaceae), *Armeniaca sibirica* (Rosaceae), *Juglans mandshurica* (Juglandaceae), *Fraxinus rhynchophylla* (Oleaceae), *Rhamnus* sp. (Rhamnaceae), and *Vitex negundo* (Lamiaceae) (Figure 8a,b). The habitat also supported active dipteran insects belonging to Chironomidae, Sciaridae, Heleomyzidae, and Trichoceridae.

Field observations revealed that all confirmed flying individuals were males, recorded singly rather than in aggregations. Flight activity was observed only around midday, with individuals typically flying up and down at approximately 0.5 m above the ground within or near shrub thickets, seldom ascending higher, and occurring regardless of the presence of snow. All adults were captured during the day using hand nets; no individuals were attracted to light traps at night in February, and virtually no other insects were observed at the light during this period.

Marsh Tits (*Poecile palustris* (Linnaeus, 1758)) (Figure 8g) and other birds were observed perching on roadside branches and opportunistically preying on adult individuals of the new species as they flew along forested slopes. We observed tits capturing moths in mid-air and subsequently perching to consume them, removing the heads and wings, which were then scattered on the snow. These remains gradually sank into the snow surface under sunlight (Figure 8c–f). This phenomenon highlights a critical ecological energy transfer in winter forest ecosystems: during periods of extreme food scarcity, adult Lepidoptera serve as an important protein source for insectivorous birds. Such interactions emphasize the functional role of the winter fauna composed of overwintering and freshly emerged adults as foundational components of the winter food web, maintaining energy flow from lower trophic levels (moths) to higher-level consumers (birds) even under thermally constrained conditions.

In addition, the deep reddish-brown ground color of the forewings, overlaid with contrasting pale markings, likely serves as camouflage against the exposed soil, leaf litter, and bark characteristic of forest floors and tree trunks during late-winter thawing periods. This cryptic coloration may function through background matching and disruptive patterning, reducing detectability by visually hunting predators such as birds. Similar wing pattern morphology is also observed in other Hadeninae genera, such as *Perigrapha* and *Lasianobia*.

## 4. Discussion

### 4.1. Taxonomic Status and Classification of Orthosiini

To better understand the systematic position of *Shoudus* gen. nov., it is necessary to clarify the taxonomic framework of the tribe Orthosiini. Historically, the tribe has been placed within the subfamily Hadeninae of Noctuidae (Ronkay et al., 2010 [6]). However, some recent studies have adopted a ‘lumping’ concept, in which all groups formerly treated as Noctuinae, Hadeninae, and Xyleninae are combined into a single, broadly defined subfamily Noctuinae, and Orthosiini is treated as a tribe within this expanded subfamily (e.g., Lafontaine and Schmidt, 2010; Kononenko and Pinratana, 2013; Han et al., 2020; Keegan et al., 2021, etc. [26,27,28,29]). In our view, under this ‘lumping’ concept Orthosiini can no longer be regarded as a tribe but should instead be classified as the subtribe Orthosiina. More importantly, consistent with the descriptions by Ronkay et al. (2001) [7] and Fibiger and Lafontaine (2005) [30], the Hadeninae possess distinctly hairy eyes (interfacetal hairs), which serves as a definitive diagnostic character for this subfamily, whereas the Noctuinae typically have smooth eyes. We concur with the view that these two lineages should be treated as independent subfamilies based on this stable morphological differentiation. Accordingly, in the present study we follow the traditional ‘splitting’ concept and treat Orthosiini as a tribe within Hadeninae.

Most species in Orthosiini are univoltine and arboricolous, inhabiting forested ecosystems ranging from lowland and hilly zones to mid-elevation and montane forests. Adults typically fly in early spring or late autumn, and the larvae feed on a wide variety of broadleaved and coniferous trees as well as shrubs, pupating and overwintering in strong cocoons in the soil (Ronkay et al., 2001 [7]). Members of Orthosiini are best characterized by the structure of the larval hypopharynx (Fibiger and Lafontaine, 2005 [30]). Although the larval stage of *Shoudus* is currently unknown, its tribal placement is supported by adult morphological characters and confirmed by molecular phylogenetic analysis, which consistently nests the new genus within the Orthosiini clade. Orthosiini exhibits remarkably high species richness and endemism in the wide sense Himalayan region, particularly across the Himalayan-Sino-Pacific region (Ronkay et al., 2010 [6]). Recent taxonomic studies in Asia have revealed numerous undescribed species and even several new genera, highlighting the underexplored nature of this group (e.g., Ronkay et al., 2010 [6]; Gyulai et al., 2011 [31]; Poorshabanan and Shirvani, 2022 [32], etc.).

### 4.2. Morphology and Phlyogeny

The placement of *Shoudus* within the Hadeninae: Orthosiini is corroborated by several diagnostic morphological characters defined by Fibiger and Lafontaine (2005) [30]. Most notably, the eyes of the new genus are densely covered with fine hairs, an ‘immensely useful’ character in associating the vast majority of Hadeninae taxa and distinguishing them from the typically smooth-eyed Noctuinae. Additionally, the lack of sclerotized tibial spines and the characteristic late-winter/spring phenology further align the new genus with Orthosiini, distinguishing it from the morphologically similar but autumn-flying Xylenini. While some genital features in *Shoudus* exhibit extreme simplification, its structural ground plan and ecological traits provide a robust morphological basis for its current tribal assignment.

Morphologically, *Shoudus* differs markedly from other known Orthosiini genera by its smaller body size, uniformly colored wings, long and sparsely bipectinate male antennae, the reduced proboscis, and simplified male genitalia with unmodified valvae. These characters are uncommon among described genera of the tribe and can serve as reliable diagnostic features at the generic level. The partial resemblance between the male genitalia of the new genus and those of *Himalaea* (Amphipyrinae: Psaphidini) and *Ulochlaena* (Xyleninae: Episemini) is likely attributable to convergent evolution or the retention of plesiomorphic traits. However, a rigorous comparative analysis reveals fundamental structural divergences that clarify its taxonomic placement. Following the diagnostic criteria for Orthosiini (Ronkay et al., 2001 [7]), *Shoudus* possesses distinctly hairy eyes and a the harpe-ampulla complex are present, the ampullar process is regularly larger, erect from the valval plane, not fused with the costal extension or the pollex—traits that are characteristic of the Orthosiini ground plan. In contrast, *Ulochlaena* lacks interfacetal hairs, and its genital configuration is defined by a flattened ampulla typically fused with the costal extension, alongside a dominant, erect harpe.

This deep evolutionary separation is further confirmed by our molecular data: the K2P genetic distance between the new genus and *H. unica* ranges from 14.55% to 15.69%, while the distance to *U. hirta* is 10.76%. These high values, far exceeding typical intergeneric thresholds, clearly support the placement of the new genus within Orthosiini, as corroborated by both the categorical morphological distinctions and molecular phylogenetic analyses.

Within the tribe, the new genus forms a well-supported clade with *Orthosia*, suggesting a relatively close evolutionary relationship. However, due to limited taxon sampling—particularly among other Orthosiini genera—and the low support values at some deeper nodes in the COI-based trees, we refrain from overinterpreting the intergeneric relationships based solely on mitochondrial data. Future studies should incorporate additional nuclear markers (e.g., EF-1α, CAD) or genome-scale data and expand taxon sampling within Hadeninae to more robustly resolve the phylogenetic position and evolutionary history of this newly established genus.

It should be noted that the current description of *Shoudus* is based solely on male specimens. Due to the extreme environmental conditions and unique phenology of this late-winter lineage, females and immature stages remain unknown. Future surveys are required to provide a more comprehensive understanding of its life history.

### 4.3. Host Plant Associations

*Orthosia* species are known to feed predominantly on *Quercus* (Fagaceae) in Japan, while in the Nearctic and Palaearctic regions, their host range extends to Rosaceae, Salicaceae, Pinaceae, Juglandaceae, and Aceraceae. The genus *Perigrapha* is generally considered polyphagous, with larvae feeding on various herbs and woody plants, specific associations with Fagaceae have been documented. For instance, *Perigrapha hoenei* has been recorded feeding on *Quercus*, and *Clavipalpula aurariae* is known to utilize both *Quercus* and *Castanea* as larval hosts (Robinson et al., 2023 [33]). Given that the Baihuashan reserve is characterized by temperate mixed forests dominated by Fagaceae, Pinaceae, Juglandaceae, and Aceraceae (Ma and Liu, 2003 [34]), it is reasonable to infer that Fagaceae, particularly *Quercus*, are the most likely primary larval hosts for the new genus. Other possible secondary hosts may include the taxa from Rosaceae, Juglandaceae, Pinaceae, and Aceraceae, all of which are also present in the reserve. Nevertheless, definitive host plant associations require further evidence through larval rearing or direct field observations.

### 4.4. Adult Biology and Overwintering Strategy

Biologically, our field observations raise uncertainty regarding the overwintering strategy of *Shoudus*: is this genus truly a moth group that overwinters as adults? Two possibilities could explain the observations. One is that adults might emerge in the late autumn but not or only exceptionally fly before the winter, and the overwhelming majority is active only at the end of the winter (or, even, in the winter period). The other possibility is that adults may enclose in late winter and are active immediately after their wings dried out. Based on the following evidence, we hypothesize the latter scenario may be more likely. The absence of functional proboscis in males suggests that they do not feed; their diurnal activity further implies that they are searching for females and not for food. Moreover, if they were to overwinter as adults without a usable proboscis, they would likely possess large fat bodies in the abdomen, as in *Dasypolia* Guenée, 1852. However, this is not the case. Instead, the dense pubescence and lack of feeding structures suggest they are ephemeral adults with a limited lifespan. In addition, the observed low-altitude flight of males suggests a potential search pattern for females sitting close to the ground—similar to the behavior of many day-flying lasiocampids and also certain noctuids. Taken together, these inferences suggest that *Shoudus* is likely a genus that emerge in late winter or early spring and take flight quickly under cold and snowy conditions, rather than one that overwinters as adults.

### 4.5. Ecological and Adaptive Significance

Apart from its taxonomic significance, our field observations reveal the potential ecological importance of *Shoudus* as a rare representative of noctuid moths active during late winter in northern China. As one of the few insect lineages active during the food-scarce late winter period, *Shoudus* may serve as an important nutritional link for insectivorous birds such as the Marsh Tit. Insect diversity in northern forested areas is extremely limited during the transition from late winter to early spring, and winter-active adults like those in the newly discovered genus in this study may play a key role in sustaining early spring food webs for forest birds. We advocate for the establishment of high-temporal-resolution monitoring networks of insect–bird trophic interactions to better understand how such basal resources support forest ecosystems during early spring.

The forewing coloration of this new species may also have ecological significance, particularly in providing camouflage against the late-winter background of snow cover and leafless vegetation. Similar cryptic features are also observed in some other Hadeninae species. Whether the high-contrast forewing markings of *S. baihuashanus* sp. nov. function in background matching or disruptive patterning remains unclear. However, its relatively high activity under late-winter low-temperature conditions may indicate a degree of thermoregulatory capability or other physiological adaptations. To more fully understand the evolutionary position and ecological adaptations of this newly discovered lineage, future work should integrate broader ecological sampling and multilocus phylogenetic analyses.

## Figures and Tables

**Table 1:** Sampling information and BOLD Sample ID/GenBank accession numbers of Orthosiini and outgroups used in this study.

Taxon	Voucher No.	Locality	GenBank No.	BOLD ID	Remark
*Shoudus baihuashanus* gen. et sp. nov. *	HHL24_001 (HT)	Beijing, China	PX069813	–	this study
*S. baihuashanus* gen. et sp. nov. *	HHL24_002 (PT)	Beijing, China	PX069814	–	this study
*S. baihuashanus* gen. et sp. nov. *	HHL24_003 (PT)	Beijing, China	PX069815	–	this study
*Clavipalpula aurariae* *	BC ZSM Lep 34670	Taiwan, China	HQ957750	GWORZ324-10	–
*Hadena eximia*	HHL24_027	Qinghai, China	PX069819	–	this study
*Lasianobia fickleri*	HHL24_038	Xizang, China	PX069823	–	this study
*L. fickleri*	HHL24_039	Xizang, China	PX069824	–	this study
*L. fickleri*	HHL24_040	Xizang, China	PX069825	–	this study
*L. dichelostigma*	HHL24_036	Xizang, China	PX069821	–	this study
*L. dichelostigma*	HHL24_037	Xizang, China	PX069822	–	this study
*L. superba*	Las1Tibet	Xizang, China	MH722409	–	–
*Niaboma xena* *	HHL24_022	Qinghai, China	PX069818	–	this study
*Orthosia incerta* *	KLM Lep 01545	Kaernten, Austria	–	ABOLA025-14	–
*Perigrapha i-cinctum* *	TLMF Lep 20119	Niederoesterreich, Austria	–	ABOLC086-16	–
*P. sechuana*	HHL24_035	Xizang, China	PX069820	–	this study
*Himalaea unica* *	LS0909030M	Xizang, China	JX392408	–	out group
*H. unica* *	HHL24_014	Xizang, China	PX069816	–	this study/out group
*H. unica* *	HHL24_020	Xizang, China	PX069817	–	this study/out group
*Ulochlaena hirta* *	MM27345	Orenburgskaya Oblast, Russia	–	LEFIJ21336-21	out group

“HT” means holotype; “PT” means paratype; “–” means no available sequences from GenBank or BOLD; “*” means the type species of each genus.

**Table 2:** Pairwise K2P distances of COI sequences for new species and some selected known species used in this study.

Species Code	Voucher No.	Taxon	1	2	3	4	5	6	7	8	9	10
1	HHL24-001 (HT)	*Shoudus baihuashanus* gen. et sp. nov.										
2	HHL24-002 (PT)	*S. baihuashanus* gen. et sp. nov.	**0.003**									
3	HHL24-003 (PT)	*S. baihuashanus* gen. et sp. nov.	**0.003**	**0.000**								
4	ABOLA025-14	*Orthosia incerta*	**0.062**	**0.062**	**0.062**							
5	ABOLC086-16	*Perigrapha i-cinctum*	0.065	0.065	0.065	0.068						
6	HHL24-035	*P. sechuana*	0.068	0.064	0.064	0.071	0.050					
7	GWORZ324-10	*Clavipalpula aurariae*	0.077	0.077	0.077	0.080	0.061	0.069				
8	HHL24-038	*Lasianobia fickleri*	0.083	0.078	0.078	0.096	0.078	0.083	0.087			
9	HHL24-037	*L. dichelostigma*	0.085	0.080	0.080	0.098	0.076	0.085	0.085	0.023		
10	HHL24-022	*Niaboma xena*	0.088	0.088	0.088	0.106	0.089	0.105	0.106	0.088	0.090	
11	HHL24-027	*Hadena eximia*	**0.097**	**0.097**	**0.097**	0.095	0.082	0.082	0.110	0.083	0.083	0.092

“HT” means holotype; “PT” means paratype; intraspecific distance of the new species, maximum and minimum interspecific distances between the new species and the compared species, are highlighted in bold.

**Table 3:** Field records of *S. baihuashanus* sp. nov. in Baihuashan National Nature Reserve, Beijing.

No.	Date	Location	Coordinates	Elevation (m)	Temperature (°C)
1	8 March 2016	Baihuashan, Qingshui Town, Mentougou District, Beijing	39°50′23.21″ N, 115°34′27.75″ E	1142	−2~6
2	19 February 2023	Malan Village, Zhaitang Town, Mentougou District, Beijing	39°55′36.06″ N, 115°41′9.87″ E	690	−3~11
3	7 February 2024	Baihuashan, Qingshui Town, Mentougou District, Beijing	39°50′23.21″ N, 115°34′27.75″ E	1142	−7~6

## Data Availability

The original contributions presented in this study are included in the article. Further inquiries can be directed to the corresponding author.

## References

[1] Kirk D.A., Evenden M.D., Mineau P., Nolan V., Ketterson E.D. (1996). Past and Current Attempts to Evaluate the Role of Birds as Predators of Insect Pests in Temperate Agriculture. Current Ornithology.

[2] Yazdanian M., Kankaanpää T., Merckx T., Huikkonen I.-M., Itämies J., Jokimäki J., Lehikoinen A., Leinonen R., Pöyry J., Sihvonen P. (2024). Evidence for bottom-up effects of moth abundance on forest birds in the north-boreal zone alone. Ecol. Lett..

[3] Stavenga D.G., Wallace J.R., Warrant E.J. (2020). Bogong moths are well camouflaged by effectively decolourized wing scales. Front. Physiol..

[4] Kang C., Stevens M., Moon J.-Y., Lee S.-I., Jablonski P.G. (2015). Camouflage through behavior in moths: The role of background matching and disruptive coloration. Behav. Ecol..

[5] Walton O.C., Stevens M. (2018). Avian vision models and field experiments determine the survival value of peppered moth camouflage. Commun. Biol..

[6] Ronkay G., Ronkay L., Gyulai P., Hacker H. (2010). New Orthosiini (Lepidoptera, Noctuidae, Hadeninae) species and genera from the wide sense Himalayan region. Esperiana.

[7] Ronkay L., Yela J.L., Hreblay M. (2001). Noctuidae Europaeae. Vol. 5, Hadeninae II.

[8] Saldaitis A., Benedek B., Volynkin A.V. (2022). *Himalaea batanga*, a new species from Southwest China (Lepidoptera: Noctuidae: Amphipyrinae: Psaphidini). Ecol. Montenegrina.

[9] Saldaitis A., Ivinskis P., Borth R. (2012). Two new *Perigrapha* species from China (Lepidoptera, Noctuidae). Zootaxa.

[10] Saldaitis A., Volynkin A.V., Truuverk A. (2018). Three new species of the genus *Lasianobia* Hampson, 1905 (Lepidoptera, Noctuidae) from China, with a revised checklist for the genus. Zootaxa.

[11] Aulombard F., Landry B., Lopes-Curval P., Ronkay G., Ronkay L., Varga Z., Landry B. (2020). La collection Jacques Plante de Noctuidae. Première partie. Noctuinae et Hadeninae. The Plante Noctuidae Collection. Part 1. Noctuinae and Hadeninae. Mémoires de la Société de Physique et d’Histoire Naturelle de Genève.

[12] Wiesmair B., Shirvani A., Ronkay L. (2020). A new *Orthosia* Ochsenheimer, 1816 species from Iran (Lepidoptera, Noctuidae, Hadeninae). Nota Lepidopterol..

[13] Gyulai P., Ronkay L. (2001). The Noctuidae material collected by Péter Gyulai & Adrienne Garai in the Qinghai region, China, in 1999 (Lepidoptera). Esperiana.

[14] Staudinger O. (1896). Beschreibungen neuer Lepidopteren aus Tibet. Dtsch. Entomol. Z. Iris.

[15] Kononenko V.S., Han H.L. (2007). Atlas Genitalia of Noctuidae in Korea (Lepidoptera). Insects of Korea Series 11.

[16] Folmer O., Black M., Hoeh W., Lutz R., Vrijenhoek R. (1994). DNA primers for amplification of mitochondrial cytochromec c oxidase subunit I from diverse metazoan invertebrates. Mol. Mar. Biol. Biotechnol..

[17] Katoh K., Standley D.M. (2013). MAFFT multiple sequence alignment software version 7: Improvements in performance and usability. Mol. Biol. Evol..

[18] Zhang D., Gao F., Jakovlić I., Zou H., Zhang J., Li W.X., Wang G.T. (2020). PhyloSuite: An integrated and scalable desktop platform for streamlined molecular sequence data management and evolutionary phylogenetics studies. Mol. Ecol. Resour..

[19] Capella-Gutiérrez S., Silla-Martinez J.M., Gabaldon T. (2009). trimAl: A tool for automated alignment trimming in large-scale phylogenetic analyses. Bioinformatics.

[20] Nguyen L.T., Schmidt H.A., von Haeseler A., Minh B.Q. (2015). IQ-TREE: A fast and effective stochastic algorithm for estimating maximum-likelihood phylogenies. Mol. Biol. Evol..

[21] Minh B.Q., Nguyen M.A., von Haeseler A. (2013). Ultrafast approximation for phylogenetic bootstrap. Mol. Biol. Evol..

[22] Anisimova M., Gil M., Dufayard J.F., Dessimoz C., Gascuel O. (2011). Survey of branch support methods demonstrates accuracy, power, and robustness of fast likelihood-based approximation schemes. Syst. Biol..

[23] Guindon S., Dufayard J.F., Lefort V., Anisimova M., Hordijk W., Gascuel O. (2010). New algorithms and methods to estimate maximum-likelihood phylogenies: Assessing the performance of PhyML 3.0. Syst. Biol..

[24] Ronquist F., Teslenko M., van der Mark P., Ayres D.L., Darling A., Höhna S., Larget B., Liu L., Suchard M.A., Huelsenbeck J.P. (2012). MrBayes 3.2: Efficient Bayesian phylogenetic inference and model choice across a large model space. Syst. Biol..

[25] Kumar S., Stecher G., Suleski M., Sanderford M., Sharma S., Tamura K. (2024). MEGA12: Molecular Evolutionary Genetics Analysis version 12 for adaptive and green computing. Mol. Biol. Evol..

[26] Lafontaine D., Schmidt C. (2010). Annotated check list of the Noctuoidea (Insecta, Lepidoptera) of North America north of Mexico. ZooKeys.

[27] Kononenko V.S., Pinratana A. (2013). Moths of Thailand, Vol. 3, Part 2. An Illustrated Catalogue of Erebidae, Nolidae, Euteliidae and Noctuidae (Insecta, Lepidoptera) in Thailand (Insecta, Lepidoptera).

[28] Han H.L., Kononenko V.S., Li C.D. (2020). The Catalogue of the Noctuoidea in the Three Provinces of Northeast China I: Families Erebidae (Part), Euteliidae, Nolidae and Noctuidae.

[29] Keegan K.L., Rota J., Zahiri R., Zilli A., Wahlberg N., Schmidt B.C., Lafontaine J.D., Goldstein P.Z., Wagner D.L. (2021). Toward a Stable Global Noctuidae (Lepidoptera) Taxonomy. Insect Syst. Divers..

[30] Fibiger M., Lafontaine J.D. (2005). A review of the higher classification of the Noctuoidea (Lepidoptera) with special reference to the Holarctic fauna. Esperiana.

[31] Gyulai P., Ronkay L., Saldatis A. (2011). New Noctuidae species from China and the Himalayas (Lepidoptera, Noctuoidea) (plates 23–26). Esperiana Band.

[32] Poorshabanan P., Shirvani A. (2022). A catalogue of Orthosiini Guenée (Lepidoptera, Noctuidae, Hadeninae) of Iran, with a new species record. J. Insect Biodivers. Syst..

[33] Robinson G.S., Ackery P.R., Kitching I., Beccaloni G.W., Hernández L.M. (2023). HOSTS—A Database of the World’s Lepidopteran Hostplants [Data Set].

[34] Ma J., Liu Q. (2003). Flora of Beijing: An overview and suggestions for future research. Urban Habitats.

